# Prognostic value of ESR2 expression on adjuvant chemotherapy in completely resected NSCLC

**DOI:** 10.1371/journal.pone.0243891

**Published:** 2020-12-17

**Authors:** Hongliang Yu, Dayong Gu, Pudong Qian

**Affiliations:** Department of Radiation Oncology, Jiangsu Cancer Hospital & Jiangsu Institute of Cancer Research & The Affiliated Cancer Hospital of Nanjing Medical University, Nanjing, Jiangsu, People’s Republic of China; Shantou University Medical College, CHINA

## Abstract

**Background:**

Prognostic biomarker, which can inform the treatment outcome of adjuvant chemotherapy (ACT) after complete resection of early-stage non-small cell lung cancer (NSCLC), is urgently needed for the personalized treatment of these patients.

**Patients and methods:**

The prognostic value of gene expression of the estrogen receptor (ER) on the effect of ACT in completely resected NSCLC was investigated in the present study. Two independent datasets from Gene Expression Omnibus (GEO) with a total of 309 patients were included in this study. The prognostic value of ER gene expression on ACT’s efficacy was evaluated by survival analysis and Cox hazards models.

**Results:**

We found a consistent and significant prognostic value of ERβ (ESR2) expression for ACT’s efficacy in completely resected NSCLC in both of the two independent cohorts. After multivariate adjustment, a significant survival benefit of ACT was observed in patients with low expression of ESR2, with a hazard ratio (HR) of 0.19 (95%CI 0.05–0.82, p = 0.026) in the discovery cohort and an HR of 0.27 (95%CI 0.10–0.76, p = 0.012) in the validation group. No significant benefit of ACT in the subgroup of patients with high expression of ESR2 was observed, with an HR of 0.80 (95%CI 0.31–2.09, p = 0.644) in the discovery cohort and an HR of 1.05 (95%CI 0.48–2.29, p = 0.896) in the validation group.

**Conclusion:**

A significant survival benefit from ACT was observed in patients with low ESR2 expression. No significant survival benefit was observed in patients with high ESR2 expression. Detection of ESR2 expression in NSCLC may help personalize its treatment after complete resection.

## Introduction

Lung cancer is the leading lethal cancer worldwide, and non-small cell lung cancer (NSCLC) accounts for more than 80% of all lung cancer cases [1]. A series of phase III studies has revealed the benefit of adjuvant chemotherapy (ACT) after resection of stage II-IIIA NSCLC, with a modest improvement in 5-year overall survival (OS) ranging from 4% to 15% [2–4]. For early-stage (stage IB) NSCLC, ACT showed no significant benefit for 5-year OS; however, more than 30% of these patients developed recurrence and died due to the disease [5, 6]. Therefore, considering the modest benefit and associated toxicity of ACT for early-stage NSCLC, it is vital to find a biomarker to prospectively distinguish patients who will benefit from ACT and spare those patients who are unlikely to benefit.

Recently, an increasing number of studies focused on the potential roles of sex hormones, especially estrogen signaling in pathophysiological features of NSCLC were published. Evidence from both in vivo and in vitro studies has shown that NSCLC is primarily an estrogen receptor (ER) positive tumor type. Histological studies of patient tissue have shown that both isoforms of ERs (ERα and ERβ) were expressed in NSCLC tumors [7]. Evidence from the cytological study also showed that both estrogen receptor genes ESR1 and ESR2, which encode ERα and ERβ, respectively, were expressed in a majority of human NSCLC cell lines [8]. Of the two isoforms of ERα and ERβ, significant differences in the pattern of expression and biological significance were observed. ERβ is the dominant isoform and was expressed in about 90% of tumor specimens in NSCLC, while ERα expression is generally low [9, 10]. Furthermore, the biological effects of estrogen signaling are known to be primarily mediated by ERβ. Evidence has shown that in lung cancer cells, ERβ is sufficient to induce the full range of estrogenic responses. Cytoplasmic ERβ expression was identified as an independent negative prognostic predictor of time to progression. ERα was elevated in NSCLC but was not predictive of survival. It is expressed not as an intact fully functional protein but as variant protein that only can be recognized by the C-terminal antibody [7, 10, 11]. These studies have provided robust evidence on the links between ER signaling and pathophysiological features of NSCLC, indicated the prognostic effect of ERs expression on NSCLC. However, to date, most studies [7, 12–15] have focused on the relationships between ER expression and survival prognosis; very few studies investigate the influence of ER expression status on the outcome of treatment for NSCLC, which may contribute to establishing ER expression as a useful biomarker for clinical treatment decision. To investigate the possible influence of ER expression on the effect of ACT in NSCLC, we searched the GEO database and reported here the microarray data-based evidence of the predictive effect of ER gene expression on ACT’s efficacy in patients with completely resected NSCLC.

## Patients and methods

### Data searching strategy

We searched the PubMed GEO database by using the following keywords: lung cancer, NSCLC, estrogen receptor, and adjuvant chemotherapy. The publications returned by the search were screened for public availability of microarray expression data and matched clinical and follow-up data. Finally, we obtained two independent studies that satisfied the criteria. The study of Tang *et al*. [16] was used as the discovery study because it has more number of cases included in the dataset, and the study of Zhu *et al*. [17] was used as the validation set.

### Discovery set

A total of 176 patients in the discovery dataset underwent curative resection for NSCLC at the MD Anderson Cancer Center between December 1996 and June 2007; patients with radiation therapy were excluded from the dataset. In this cohort, 49 patients received ACT, which mainly was carboplatin plus taxanes regimen, and 127 patients did not receive ACT. Gene expression of all samples was analyzed on the Illumina Human-6 V3 platform. Microarray data were preprocessed using GCRMA normalization with the PM-MM data. All the clinical information and gene expression data for these patients were deposited in the GEO database (GSE42127) [16].

### Validation set

A total of 133 cases were included in the validation set. All cases in this dataset were selected from the JBR.10 trial, which was a randomized controlled trial that compared adjuvant vinorelbine/cisplatin treatment with observation alone. Gene expression profiling was performed by using the U133A oligonucleotide microarrays (Affymetrix, Santa Clara, CA). Of patients with microarray profiles, 62 were in the observation (OBS) group, while 71 received ACT. All the clinical information and gene expression data for these patients were deposited in the GEO database (GSE14814) [17] and briefly presented in Table 1.

**Table 1:** Patient characteristics of both discovery and validation cohorts, grouped by high and low expression of ESR2.

	Discovery cohort (n = 176)					Validation cohort (n = 133)				
	ESR2 low (n = 88)	ESR2 high (n = 88)		ESR2 low (n = 66)	ESR2 high (n = 67)	
	n	%	N	%	p-value	n	%	N	%	p-value
SEX					0.365					0.459
male	43	48.9	50	56.8		43	65.2	48	71.6	
female	45	51.1	38	43.2		23	34. 8	19	28.4	
AGE					0.069					0.856
<65	33	37.5	46	52.3		44	66.7	43	64.2	
≥65	55	62.5	42	47.7		22	33.3	24	35.8	
PATHOLOGY					0.726					0.001**
squamous cell carcinoma	20	22.7	23	26.1		16	24.2	36	53.7	
adenocarcinoma	68	77.3	65	73.9		46	69.7	25	37.3	
other	0	0.00	0	0.00		4	6.1	6	9.0	
STAGE*					0.466**					
I	59	67.8	53	60.2		41	62.1	32	47.8	0.118
II	13	14.9	19	21.6		25	37.9	35	52.2	
III	14	16.1	16	18.2		0	0	0	0	
IV	1	1.1	0	0.00		0	0	0	0	
TREATMENT					0.737					0.935
observation	65	73.9	62	70.5		31	47.0	31	42.3	
adjuvant chemotherapy	23	26.1	26	29.5		35	53.0	36	53.7	

* Stage information was missed in 1 case

** p-values were derived from Fisher’s exact test.

### Statistical analysis

Microarray datasets and matched clinical and follow-up information was processed using Microsoft Excel software, version 2007. Statistical analysis was performed using SPSS software, version 18 (SPSS, Chicago, IL). Chi-square tests or Fisher’s exact tests were performed to determine the differences in the distribution of clinical characteristics between high and low gene expression groups for ESR1 and ESR2, respectively. Survival rates were estimated using the Kaplan–Meier method and the log-rank test. The prognostic values of ESR1 and ESR2 status on ACT were studied using a Cox model, which was adjusted for significant and available prognostic factors of survival, including age, gender, stage, and histology. All reported p values were two-sided. P values of less than 0.05 were considered statistically significant.

In the present study, the microarray dataset of GSE42127 in the discovery set was originally obtained on the Illumina Human-6 V3 platform. In the datasheet, there was one probe (ILMN_1678535) annotated to ESR1 and two probes (ILMN_1740045 and ILMN_2390457) annotated to ESR2. The microarray dataset of GSE14814 in the validation set was analyzed on the Affymetrix U133A platform. There were 9 individual probes (205225_at, 211233_x_at, 211234_x_at, 211235_s_at, 211627_x_at, 215551_at, 215552_s_at, 217163_at, and 217190_x_at) annotated to ESR1 and 5 probes (210780_at, 211117_x_at, 211118_x_at, 211119_at, 211120_x_at) annotated to ESR2. In the processing, if multiple probes corresponded to one gene, the average expression value of the probes was calculated and used as the expression value for the gene. In the present study, high gene expression was defined as gene expression above the median value, and vice versa [12, 18].

## Results

### Predictive value of ESR2 expression on the survival benefit of ACT

**Fig 1:**
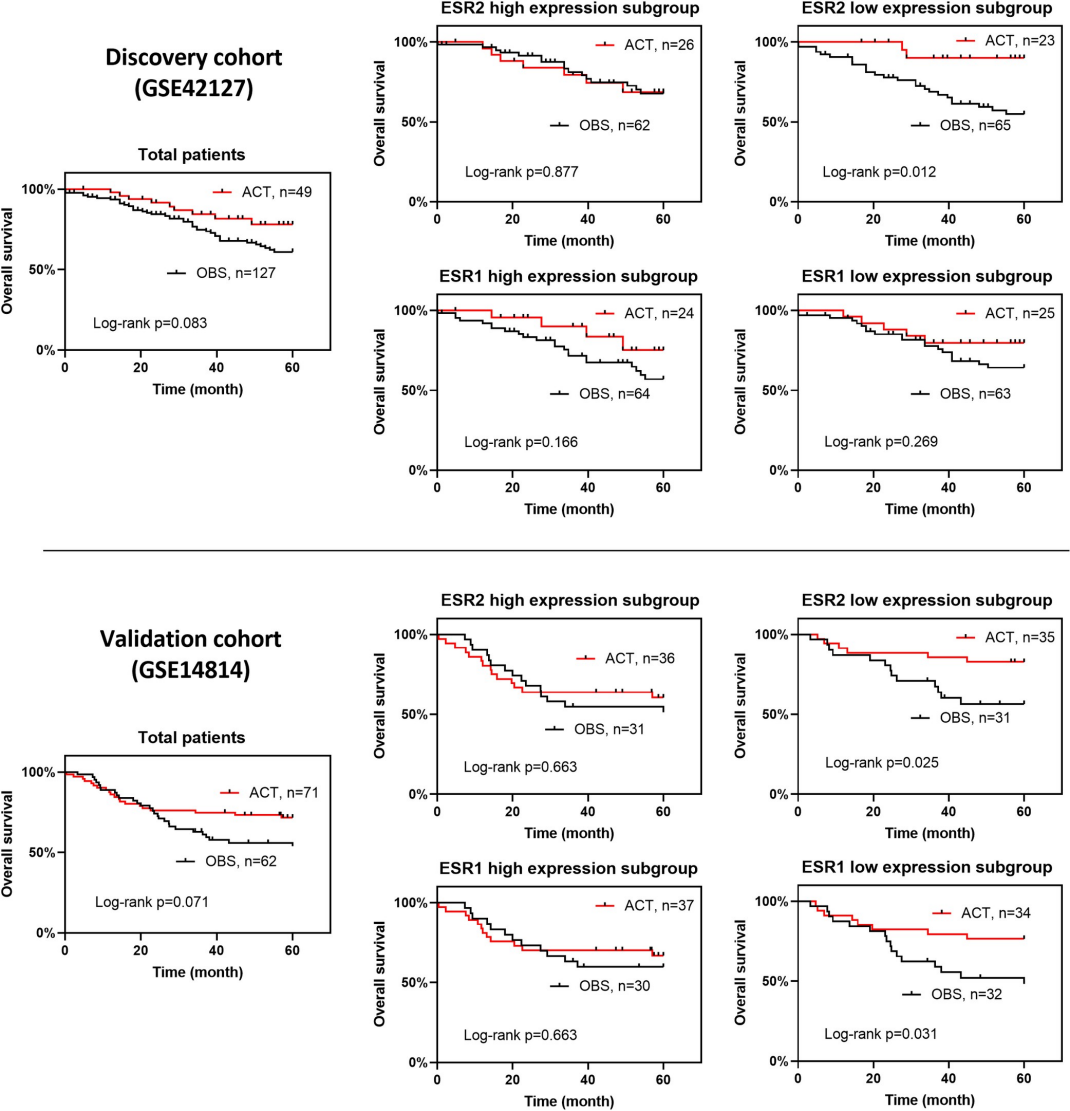
Kaplan–Meier estimates of the 5-year OS according to treatments in patients with high and low expression of ESR1 and ESR2. A consistent survival benefit of ACT in patients with low ESR2 expression after resection of NSCLC was observed in both discovery and validation sets, but the same result was not observed for ESR1. In patients with high expression of ER, regardless of ESR1 or ESR2, no survival benefit was noted in both discovery and validation sets. A, Kaplan–Meier plots of the discovery set; B, Kaplan–Meier plots of the validation set.

Kaplan–Meier estimates of the 5-year overall survival (OS) according to treatments in patients with high and low expression of both ESR1 and ESR2 are presented in Fig 1. We observed a consistent survival benefit of ACT in patients with NSCLC with low ESR2 expression after surgery in both discovery and validation sets. In the discovery set, the 5-year OS was significantly better in the ACT group than in the OBS group in patients with low ESR2 expression (log-rank p = 0.012), while patients with high ESR2 expression showed chemoresistance with no significant difference was observed in the survival rate between the ACT and OBS groups (log-rank p = 0.877). The same tendency was found in the validation set, as ACT showed a significant benefit of 5-year OS (log-rank p = 0.025) in the group with low ESR2 expression, while in patients with high ESR2 expression, no significant difference was found between the ACT and OBS groups (log-rank p = 0.663) after resection. For ESR1, although we found significant survival benefit of ACT in patients with low ESR1 expression in the validation cohort (log-rank p = 0.031), the benefit was not significant in the discovery cohort (log-rank p = 0.269), despite there was a tendency toward survival benefit of ACT. Thus, our results showed a significant predictive effect of ESR2 expression on the benefit obtained by ACT; however, the same effect was not successfully proved for ESR1. Hence, further analyses were performed with stratification factors for ESR2.

### Patient characteristics

In the discovery cohort, clinical characteristics were well balanced among patients with high and low expression of ESR2. No significant difference in age, sex, pathology, cancer stage, and treatment were noted between high and low ESR2 expression groups. In the validation cohort, patients were well balanced in terms of age, sex, cancer stage, and treatments, except for pathology, between high and low ESR2 expression groups. The high ESR2 expression group showed significantly more amount of squamous cancer (p = 0.001). The difference in the benefit of ACT between high and low ESR2 expression groups is not likely induced by the difference in pathology because a similar difference was not observed in the discovery cohort. It should be noted that a majority (278/309 = 90.0%) of the patients included in this analysis had early-stage (stage I & II) NSCLC and could receive complete resection. The details of patient characteristics are presented in Table 1.

### Cox regression analysis

For the entire patient group, in the univariate cox regression model, disease stage (late vs. early) was the only variate showed a consistent and significant predictive effect on 5-year OS, with HR of 2.00 (95% CI: 1.16–3.47, p = 0.013) and 1.94 (95% CI: 1.09–3.45, p = 0.024) in discovery and validation cohort respectively. In the multivariate cox regression model, after adjustment for age, gender, pathology, treatment, and ESR1, ESR2 expression, disease stage was still a strong prognostic factor for 5-year OS, with HR of 1.92 (95% CI: 1.08–3.40, p = 0.025) and 1.75 (95% CI: 0.97–3.15, p = 0.065) in the discovery and validation datasets, respectively. Besides, in multivariate analysis, ACT showed a marginally significant better survival, with HR of 0.49 (95% CI: 0.23–1.02, p = 0.056) and 0.56 (95% CI: 0.31–1.02, p = 0.056) in the discovery and validation datasets, respectively. When the patients were divided into high and low ESR2 expression subgroups, we observed a significant difference in the effect of ACT between the two subgroups. In high ESR2 expression subgroup, after multivariate adjustment, ACT showed no benefit on 5-year OS in both discovery and validation datasets, with HR of 0.80 (95% CI: 0.31–2.09, p = 0.644) and 1.05 (95% CI: 0.48–2.29, p = 0.896), respectively. In patients with low ESR2 expression, after adjustment for age, gender, stage, and pathology type and ESR1 expression, ACT showed a consistent and independent benefit for 5-year OS, with HR of 0.19 (95% CI: 0. 0.05–0.82, p = 0.026) and 0.27 (95%CI: 0.10–0.76, p = 0.012) in the discovery and validation datasets, respectively. The results of the Cox regression analysis are presented in Tables 2 and 3.

**Table 2:** Results of both univariate and multivariate cox regression analyses for total patients and subgroups with low and high ESR2 expression in the discovery cohort.

		Univariate			Multivariate*		
		HR	(95% CI)	p-value	HR	(95% CI)	p-value
Total patients	Age (≥65/<65)	1.36	(0.78–2.39)	0.282	1.09	(0.60–1.99)	0.769
	Gender (male/female)	1.44	(0.82–2.53)	0.203	1.36	(0.75–2.47)	0.304
	Stage (II, III, IV/I)	2.00	(1.16–3.47)	0.013	1.92	(1.08–3.40)	0.025
	Pathology (squa+other/adeno)	1.35	(0.75–2.45)	0.315	1.14	(0.62–2.08)	0.677
	Treatment (ACT/OBS)	0.53	(0.26–1.08)	0.080	0.49	(0.23–1.02)	0.056
	ESR1 (high/low)	1.16	(0.67–2.01)	0.597	1.20	(0.68–2.11)	0.530
	ESR2 (high/low)	0.78	(0.45–1.36)	0.386	0.77	(0.44–1.36)	0.363
High ESR2	Age (≥65/<65)	1.43	(0.63–3.25)	0.394	1.30	(0.56–3.02)	0.547
	Gender (male/female)	2.40	(0.95–6.08)	0.066	2.20	(0.81–5.98)	0.123
	Stage (II,III,IV/I)	2.74	(1.18–6.33)	0.019	2.44	(1.02–5.86)	0.046
	Pathology (squa+other/adeno)	1.17	(0.48–2.84)	0.735	0.97	(0.39–2.40)	0.945
	Treatment (ACT/OBS)	1.07	(0.44–2.61)	0.877	0.80	(0.31–2.09)	0.644
	ESR1 (high/low)	1.00	(0.44–2.26)	0.994	1.19	(0.50–2.85)	0.694
Low ESR2	Age (≥65/<65)	1.23	(0.56–2.72)	0.608	0.85	(0.35–2.07)	0.727
	Gender (male/female)	1.04	(0.49–2.18)	0.926	0.98	(0.45–2.17)	0.966
	Stage (II, III, IV/I)	1.61	(0.76–3.41)	0.211	1.52	(0.70–3.34)	0.293
	Pathology (squa+other/adeno)	1.56	(0.71–3.45)	0.272	1.36	(0.60–3.09)	0.466
	Treatment (ACT/OBS)	0.19	(0.05–0.82)	0.025	0.19	(0.05–0.82)	0.026
	ESR1 (high/low)	1.34	(0.64–2.81)	0.446	1.35	(0.60–3.02)	0.466

* In multivariate analysis, all listed covariates entered analysis.

**Table 3:** Results of both univariate and multivariate cox regression analyses for total patients and subgroups with low and high ESR2 expression in the validation cohort.

		Univariate			Multivariate*		
		HR	(95% CI)	p-value	HR	(95% CI)	p-value
Total patients	Age (≥65/<65)	1.93	(1.09–3.41)	0.024	2.28	(1.26–4.12)	0.006
	Gender (male/female)	1.64	(0.84–3.22)	0.150	1.61	(0.78–3.36)	0.201
	Stage (II/I)	1.94	(1.09–3.45)	0.024	1.75	(0.97–3.15)	0.065
	Pathology (squa+other/adeno)	0.95	(0.54–1.67)	0.849	0.46	(0.23–0.91)	0.025
	Treatment (ACT/OBS)	0.59	(0.33–1.05)	0.075	0.56	(0.31–1.02)	0.056
	ESR1 (high/low)	1.03	(0.59–1.82)	0.912	0.59	(0.29–1.21)	0.149
	ESR2 (high/low)	1.70	(0.96–3.04)	0.071	2.65	(1.21–5.81)	0.015
High ESR2	Age (≥65/<65)	2.20	(1.05–4.58)	0.036	2.39	(1.12–5.09)	0.024
	Gender (male/female)	2.11	(0.80–5.53)	0.130	2.81	(0.94–8.40)	0.065
	Stage (II/I)	1.89	(0.88–4.07)	0.103	1.82	(0.83–3.99)	0.136
	Pathology (squa+other/adeno)	0.61	(0.29–1.27)	0.186	0.33	(0.14–0.78)	0.012
	Treatment (ACT/OBS)	0.85	(0.41–1.76)	0.664	1.05	(0.48–2.29)	0.896
	ESR1(high/low)	0.85	(0.38–1.91)	0.689	0.61	(0.25–1.48)	0.273
Low ESR2	Age (≥65/<65)	1.59	(0.64–3.97)	0.316	2.11	(0.75–5.99)	0.160
	Gender (male/female)	1.15	(0.44–3.01)	0.785	1.16	(0.40–3.35)	0.780
	Stage (II/I)	1.69	(0.69–4.15)	0.256	1.85	(0.73–4.65)	0.193
	Pathology (squa+other/adeno)	1.10	(0.42–2.89)	0.848	0.66	(0.22–1.99)	0.457
	Treatment (ACT/OBS)	0.35	(0.13–0.91)	0.032	0.27	(0.10–0.76)	0.012
	ESR1(high/low)	0.54	(0.16–1.84)	0.322	0.50	(0.13–1.84)	0.294

*In multivariate analysis, all listed covariates entered analysis.

## Discussion

Although ACT was not recommended for early-stage (i.e., IB stage) NSCLC after radical resection, there were still 30% of these patients who would develop recurrence [5, 6]. The potential benefit of ACT may be compromised by the heterogeneity of gene expression background of these patients. It would be very helpful for personalized precision medicine to find a biomarker to prospectively distinguish which subgroup of patients will benefit from ACT and spare those patients who are unlikely to benefit. To date, increasing evidence has shown the important biological significance of ER signaling in NSCLC. Studies have shown that both estrogen receptors of ERα and ERβ were expressed in lung tumor tissue [19], while ERβ is the dominant ER isoform in NSCLC, expressed in about 90% of all NSCLC cases, ERα isoform expression is relatively low and expressed as variant fragment protein forms, not full-length protein, which can only be recognized by C-terminal antibody [7, 20, 21]. Several studies, including systematic meta-analyses, have shown that estrogen receptor expression both in mRNA and protein levels is closely related to the survival prognosis of NSCLC [11, 22]. However, no study yet available evaluate the possible influence of ER expression on treatment outcomes of NSCLC, especially on the effect of ACT after resection.

In this study, based on the microarray data derived evidence, we performed comprehensive analyses of the effect of mRNA expression of both ER isoforms of ERα (ESR1) and ERβ (ESR2) on treatment outcome of ACT in patients received completely resected NSCLC. We observed a significant prognostic value of ESR2 expression on the treatment effect of ACT. ACT showed a significant survival benefit in the low ESR2 expression subgroup in both discovery and validation groups. In patients with high ESR2 expression in both discovery and validation groups, ACT showed no benefit on patients’ survival after resection of NSCLC. As for ESR1, although we found significant survival benefit of ACT in patients with low ESR1 expression in the validation cohort, it failed to find the prognostic effect in the discovery cohort. To the best of our knowledge, the present study is the first report focusing on the prognostic role of the expression of estrogen receptor on the effect of ACT in completely resected NSCLC. When interpreting our results, it should be noted that as the majority of cases included in this analysis were early-stage NSCLC, we still believe that ACT after resection is a standard treatment for patients with advanced disease. Nevertheless, our interest was limited to those patients with early-stage disease and received complete resection, who may have difficulty in judging the choice of ACT. Thus, our results may help in personalized precision medicine for patients with NSCLC.

Several underlying molecular mechanisms may contribute to the chemoresistance of NSCLC with high protein ERβ expression. First, ERβ could induce cellular pro-survival signaling through Src kinase, MAPK, and AKT pathways. These signaling pathways are known to facilitate cell proliferation, prevent cell apoptosis, and promote treatment resistance [15, 23], thus inducing chemoresistance in NSCLC. Secondly, carcinoma cells acquiring a mesenchymal-like phenotype through the epithelial-mesenchymal transition (EMT) are thought to represent a population of cells with increased resistance to a variety of cytotoxic therapies. ERβ was reported as a strong EMT inducer by recruiting to the promoter of midkine and enhancing its expression [24]. Thirdly, a significant correlation of ERβ expression and multidrug resistance protein families was observed in various other cancer types [25, 26]; our previous analysis of TCGA NSCLC samples also showed a moderate but significant correlation (Spearman’s correlation coefficient 0.33, p<0.01) of ESR2 expression and multidrug resistance protein 1 (MDR1) at the mRNA level, which is a well-known inducer of chemoresistance.

Besides our findings on the effect of ESR2, there are some other gene signatures that have been established for predicting the benefit of ACT, especially the 12-gene signature and 15-gene signatures proposed by the two original studies [16, 17]. In fact, it is common in this area that there is not only one ideal gene signature that exists predicting treatment benefit or disease prognosis. Different gene signatures with similar predicting accuracy may exist according to different reasoning strategies [1]. Furthermore, our findings did not contradict other established signatures. Functional annotation showed that many of the signature genes from the original studies were nuclear proteins or transcription regulators (MDM2, ZNF236, FOSL2, HEXIM1, MYT1L, IKBKAP, HOPX, MBIP, NKX2-1, AURKA, RRM2, IFT57, and TTC37). Gene ESR2 encodes protein ERβ, which also belongs to the superfamily of nuclear receptor transcription factors, binds to a specific DNA sequence, the estrogen response element (ERE), and activates the expression of its reporter genes. Particularly, the aberration of gene expression of ESR2 significantly interacts with predominantly of the 27 signature genes from the two original studies [16, 17] (S1 Fig). These may suggest they share some common signaling pathways.

For the possible future application of protein ERβ in the treatment of NSCLC, several concerns need to be addressed. First, studies have reported the expression of ERβ in the range of 9–98% [9, 27, 28] in NSCLC; this inconsistency is possibly due to the heterogeneity in methodologies, including the antibodies used for immunohistochemical staining as well as inconsistent definitions of positivity. Therefore, for future studies, a validated antibody and a consistent definition of the threshold of high expression should be established. Second, the present study is a retrospective study based on bioinformatics data mining, and a prospective randomized trial should be performed to establish the credibility and accuracy of the prognostic effect of the gene and/or protein expression of ERβ on ACT in patients with early-stage NSCLC after resection.

## Conclusion

On the basis of microarray data evidence from two independent datasets, we found a consistent significant prognostic value of ESR2 expression on ACT in patients with completely resected early-stage NSCLC. A significant survival benefit of ACT was observed in patients with low expression of ESR2, while no benefit of ACT was noted in patients with high expression of ESR2. This may help in distinguishing patients with NSCLC who will benefit from ACT after resection, thus sparing those who will not benefit from ACT from suffering chemotherapy.

## Supporting information

S1 Fig ESR2 interacts with signature genes of the two original studies. (DOCX)
