# Supplementary material for: Prognostic value of ESR2 expression on adjuvant chemotherapy in completely resected NSCLC
Source: PLoS One. 2020 Dec 17;15(12):e0243891. doi: 10.1371/journal.pone.0243891 (PMC7746143; doi:10.1371/journal.pone.0243891)
Supplement: S1 Fig — (DOCX) [file pone.0243891.s001.docx]

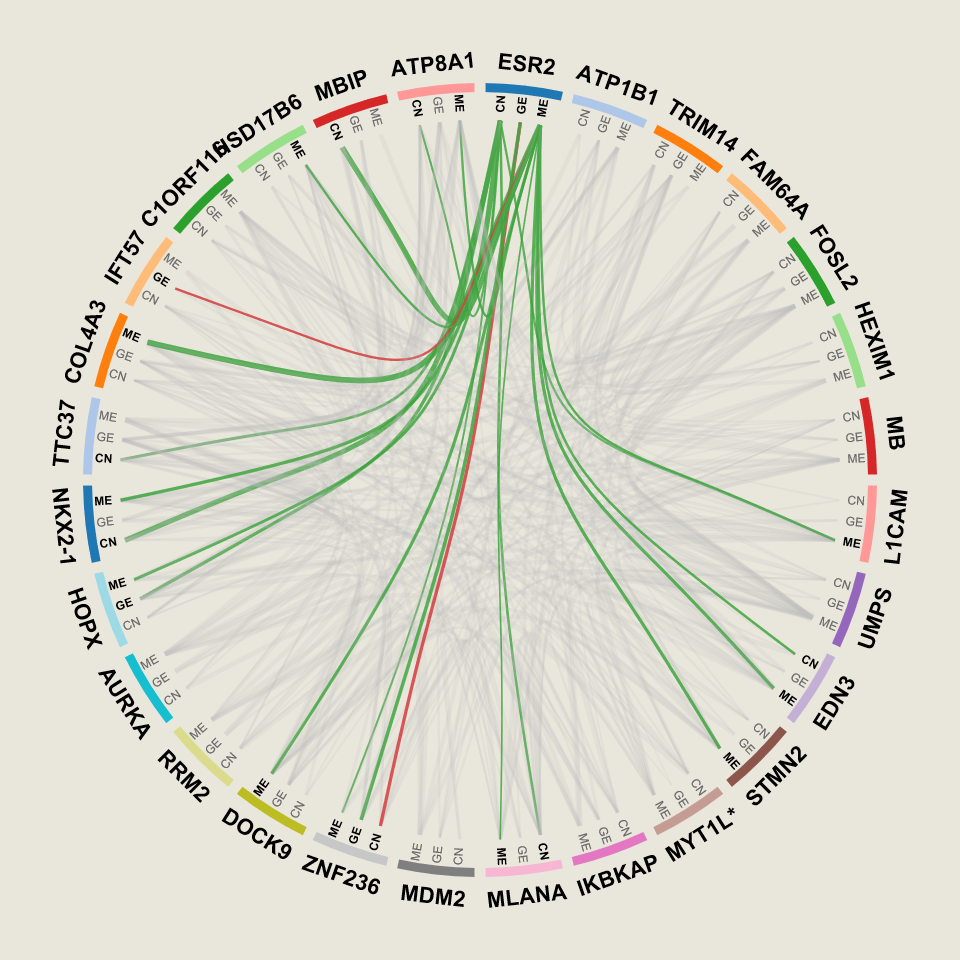


S1 Fig. ESR2 interacts with signature genes of the two original studies.

By bioinformatic research, we found the aberration of gene expression of ESR2 significantly interacts with predominance of the 27 signature genes raised in the 2 original studies. Green lines indicate positive interactions and red lines indicate negative interactions.

Abbreviations: GE = gene expression; ME= gene methylation; CN=copy number

For more detailed methodology information, reference to work of zhu et.al.(PMID: 25956356)
